# Clinical consequences of submicroscopic malaria parasitaemia in Uganda

**DOI:** 10.1186/s12936-018-2221-9

**Published:** 2018-02-05

**Authors:** Shereen Katrak, Patience Nayebare, John Rek, Emmanuel Arinaitwe, Joaniter I. Nankabirwa, Moses Kamya, Grant Dorsey, Philip J. Rosenthal, Bryan Greenhouse

**Affiliations:** 1 Department of Medicine, University of California San Francisco, San Francisco, USA; 2 Infectious Diseases Research Collaboration, Kampala, Uganda; 3 London School of Hygiene and Tropical Medicine, London, UK; 4 School of Medicine, Makerere University College of Health Sciences, Kampala, Uganda

**Keywords:** Malaria, Submicroscopic infection, LAMP, Molecular epidemiology, Clinical tropical medicine

## Abstract

**Background:**

Submicroscopic malaria parasitaemia is common in both high- and low-endemicity settings, but its clinical consequences are unclear.

**Methods:**

A cohort of 364 children (0.5–10 years of age) and 106 adults was followed from 2011 to 2016 in Tororo District, Uganda using passive surveillance for malaria episodes and active surveillance for parasitaemia. Participants presented every 90 days for routine visits (n = 9075); a subset was followed every 30 days. Participants who presented with fever and a positive blood smear were treated for malaria. At all routine visits microscopy was performed and samples from subjects with a negative blood smear underwent loop-mediated isothermal amplification for detection of plasmodial DNA.

**Results:**

Submicroscopic parasitaemia was common; the proportion of visits with submicroscopic parasitemia was 25.8% in children and 39.2% in adults. For children 0.5–10 years of age, but not adults, having microscopic and submicroscopic parasitaemia at routine visits was significantly associated with both fever (adjusted risk ratios [95% CI], 2.64 [2.16–3.22], 1.67 [1.37–2.03]) and non-febrile illness (aRR [CI], 1.52 [1.30–1.78], 1.26 [1.09–1.47]), compared to not having parasitaemia. After stratifying by age, significant associations were seen between submicroscopic parasitaemia and fever in children aged 2–< 5 and 5–10 years (aRR [CI], 1.42 [1.03–1.98], 2.01 [1.49–2.71]), and submicroscopic parasitaemia and non-febrile illness in children aged 5–10 years (aRR [CI], 1.44 [1.17–1.78]). These associations were maintained after excluding individuals with a malaria episode within the preceding 14 or following 7 days, and after adjusting for household wealth.

**Conclusions:**

Submicroscopic malaria infections were associated with fever and non-febrile illness in Ugandan children. These findings support malaria control strategies that target low-density infections.

**Electronic supplementary material:**

The online version of this article (10.1186/s12936-018-2221-9) contains supplementary material, which is available to authorized users.

## Background

Despite coordinated efforts in vector-control and improved treatment of symptomatic disease, malaria infection remains common worldwide, causing an estimated 200 million illnesses per year [1]. The use of molecular diagnostic tests such as loop-mediated isothermal amplification (LAMP) and quantitative PCR has uncovered a large reservoir of malaria parasites previously not detectable by microscopy or rapid diagnostic test (RDT) [2–4]. There is evidence that these submicroscopic infections contribute to disease transmission, with insect feeding studies demonstrating human-to-mosquito transmission in the absence of microscopically detectable parasites [5, 6]. However, the clinical relevance of submicroscopic infection is less clear. Much of the data on submicroscopic infection are from single cross-sectional surveys, in which the low frequency of clinical outcomes may limit the statistical power to study these outcomes. Furthermore, molecular detection of low-level parasitaemia may represent early detection of replicating parasites that will ultimately result in clinical disease, or detection of gametocytes after successful treatment, and distinguishing these possibilities is not possible without longitudinal data. Although there is some evidence to suggest that low-density, chronic infections can be accompanied by clinical signs and symptoms, with associations suggested between submicroscopic parasitaemia and anaemia, altered cognitive function, inflammatory markers, and systemic bacterial infection [4, 7, 8], few studies have included sufficient follow-up to demonstrate the clinical consequences of submicroscopic parasitaemia.

To assess associations between submicroscopic parasitaemia and clinical outcomes, repeated surveys measuring parasite prevalence were performed in a cohort of Ugandan children and adults. We hypothesized that submicroscopic malaria infection would be associated with clinical outcomes, including fever, in children.

## Methods

### Ethical approval

The study protocol was reviewed and approved by the Uganda National Council of Science and Technology (Approval Number HS 1019) and the institutional review boards of the University of California—San Francisco (Approval Number 11-05995), and Makerere University (Approval Number 2011-169). Informed consent was obtained from the parent or guardian of all participating children.

### Study site and participants

The study took place in Nagongera sub-county, Tororo District, Uganda, an area where malaria transmission has been reported as high and perennial [9], with annual entomological inoculation rates of > 300 bites per person/year in 2011–12 [10]. However, transmission intensity declined dramatically after indoor residual spraying of insecticides (IRS) was initiated in December, 2014 [11]. Samples for this study were from repeated surveys in a cohort of children (age 0.5–10 years) and adults (age ≥ 18) between August 2011 and June 2016, coinciding with three rounds of IRS in December 2014, June 2015, and December 2015.

### Study design and clinical follow-up

Cohort enrolment, follow-up, and clinical care have been described previously [4, 10]. Briefly, participants were recruited from 100 randomly selected households within the catchment area of the study health facility. All children aged 6 months to 10 years plus a primary adult caretaker in the household were enrolled. Participants agreed to come to the study clinic for any febrile illness and to avoid anti-malarial medications administered outside the study. All enrolled participants were given a long-lasting insecticidal bed net.

Participants presented to the clinic for routine visits every 90 days (every 30 days for a subset of 204 patients starting in 2015). At each routine visit, clinical interviews were performed, and blood smears and dried blood spots (DBS) were collected, regardless of symptoms. For samples with negative blood smears, DNA extracted from DBS was tested for the presence of submicroscopic parasitaemia using LAMP. Participants were invited to visit the clinic any time they were ill, and microscopy was performed when there was a reported or documented fever (temperature of ≥ 38.0 °C). Patients with reported or documented fever and a positive blood smear were treated with standard dosing of artemether–lumefantrine. Participants with asymptomatic parasitaemia were not provided anti-malarial therapy in accordance with local standard-of-care.

### Laboratory methods

Thick smears were prepared with 2% Giemsa. Two expert microscopists evaluated each smear separately, and a third resolved discrepancies. DBS were prepared by spotting approximately 25 μL of blood onto filter paper, drying completely, and storing at room temperature. DNA was extracted using Chelex, as previously described [12], yielding 200 µL of DNA extraction product. LAMP was performed using Eiken *Loopamp™ Malaria* Pan Detection Kit reaction tubes and 15 µL of extracted DNA, per manufacturer’s guidelines. The LAMP primer set targets a mitochondrial DNA sequence that is conserved in four major human malaria species [13]. LAMP reactions were assessed based on visual detection of fluorescence under an ultraviolet lamp. Each batch of 48 LAMP reactions included three controls with known *Plasmodium falciparum* densities (10 parasites/µL, 1 parasite/µL, and 0 parasite/µL), as well as one positive and one negative control from the Eiken kit.

### Data analysis

Data were analysed using STATA (version 13; STATA Corp., College Station, TX, USA). The proportion of visits with no detectable parasitaemia (negative blood smear and negative LAMP reaction), submicroscopic parasitaemia (negative blood smear and positive LAMP reaction), and microscopic parasitaemia (positive blood smear) were made for participants in different age strata. Age strata were pre-specified as <2, 2–< 5, 5–10, and ≥ 18 years, based on local epidemiology explored in prior work [4]. The primary outcomes of interest included the risk of (1) fever (reported fever in prior 24 h or temperature of ≥ 38.0 °C at routine visit), (2) documented fever (temperature of ≥ 38.0 °C at routine visit), and (3) non-febrile clinical illness (composite variable based on reported abdominal pain, anorexia, vomiting, diarrhoea, cough, headache, joint pain, muscle ache, seizure, or jaundice at routine visit) among those with microscopic, submicroscopic, and no detectable parasitaemia. Generalized estimating equation models were used to estimate associations between parasitaemia status and these three outcomes to account for repeated measures, using binomial outcomes with a log link function, exchangeable correlation structure, and robust standard error estimates. A p value < 0.05 was considered statistically significant. GEE models were first constructed based on simple categorization of parasitaemia status at routine visits. To differentiate chronic low-density infection from either new infection with expanding parasite biomass or recently treated infection, participants who developed malaria within 14 days before or 7 days after a routine visit were excluded, and separate GEE models were re-constructed for these visits. Because household wealth may be independently associated with both exposure to malaria parasites and the outcome of fever and/or clinical illness, a wealth index, categorized by tertiles, was included as a covariate. Data collection and determination of the wealth index has been previously described, and was based on two surveys: (1) a baseline household survey conducted at the time of enrolment, (2) a second household survey conducted after 24 months of follow-up in September–October 2013 [14].

## Results

### Study enrolment and parasite prevalence

**Fig. 1:**
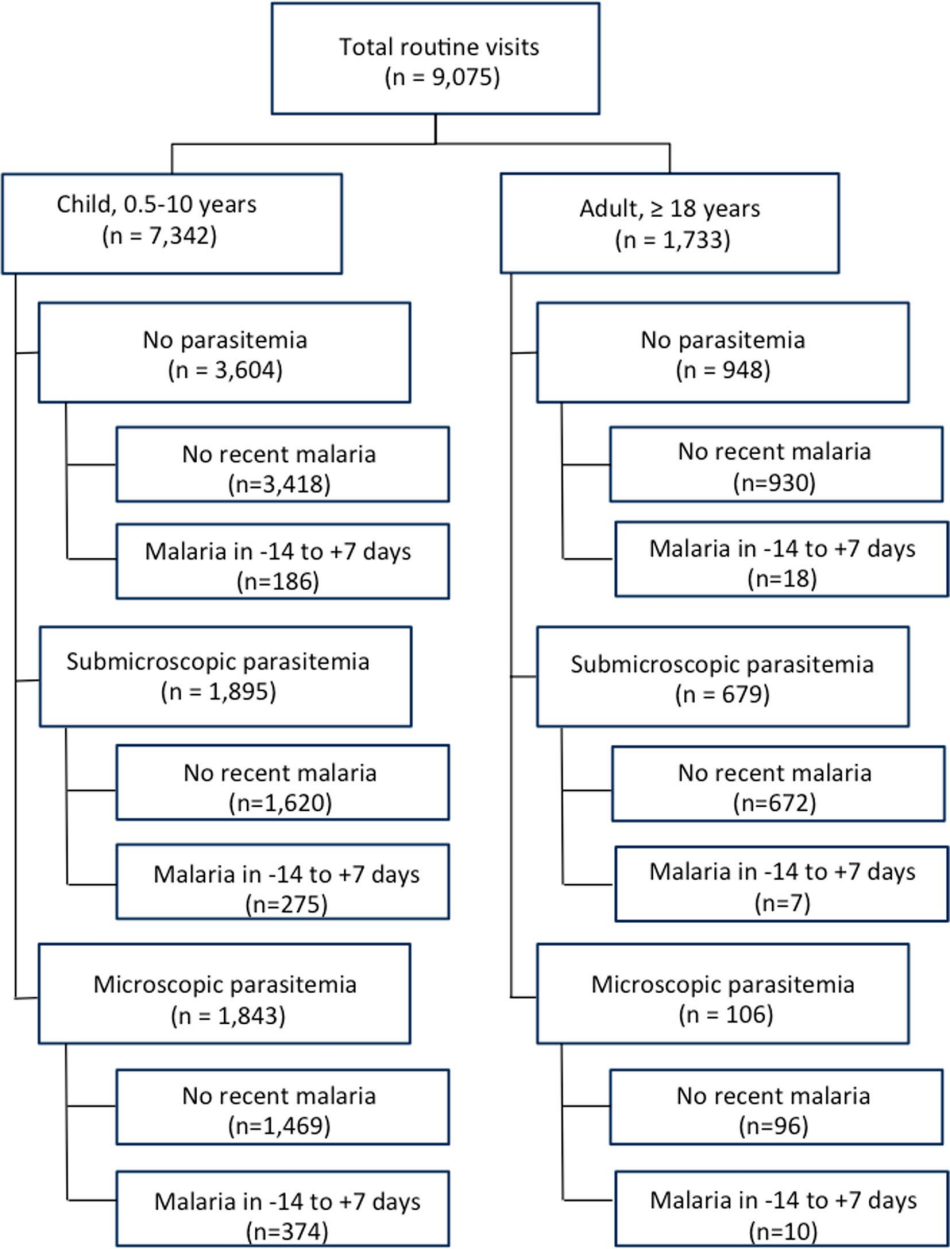
Study enrolment. The study cohort was comprised of 364 children and 106 adults, who made a total of 9075 routine visits to the study clinic. Routine visits were categorized as no parasitaemia (blood smear and LAMP negative), submicroscopic parasitaemia (blood smear negative, LAMP positive), or microscopic parasitaemia (blood smear positive). Visits were further categorized based on whether the participant had a symptomatic episode of malaria within 14 days before or 7 days following the routine visit

**Fig. 2:**
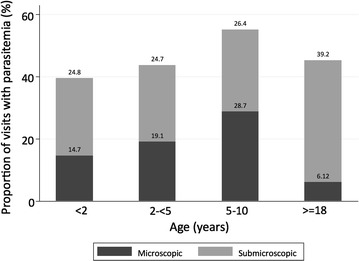
Proportion of study population with microscopic and submicroscopic parasitaemia. Participants are divided into age strata along the X axis. The percentage of positive samples, among all samples collected at routine visits for that age group, is shown on the Y axis

Over 5 years of longitudinal follow-up of 364 children and 106 adults, there were a total of 9075 routine visits, including 7342 among children 0.5–10 years, and 1733 among adults ≥ 18 years (Fig. 1). Blood smears and interviews were performed at each routine visit, and for visits with negative blood smears, blood was tested for the presence of submicroscopic parasitaemia using LAMP. Children were parasitaemic at roughly half of all routine visits, with 1843 (25.1%) microscopic infections and 1895 (25.8%) submicroscopic infections (Fig. 2). A similar proportion of adult visits were parasitaemic, but fewer had microscopic (106; 6.1%) compared to submicroscopic (679; 39.2%) infections. Further stratifying the proportion of visits with detectable parasitaemia by age revealed that microscopic parasitaemia amongst children increased with age, peaking at 28.7% among children 5–10 years old, but that submicroscopic infection remained steady, with roughly a quarter of children having submicroscopic parasitaemia across age groups.

### Association of parasitaemia and fever

In children, having microscopic parasitaemia compared to no parasitaemia was, as expected, associated with a more than twofold increase in risk of fever in all age groups (Table 1). More remarkably, in children age 2–10 years of age, submicroscopic parasitaemia was also associated with an increased risk of fever compared to those without parasitaemia (adjusted risk ratio [CI], 1.42 [1.03–1.98] for age 2–< 5 years, 2.01 [1.49–2.71] for age 5–10 years). This association was maintained for the same age range when those with recent malaria were excluded from the analysis. However, associations between submicroscopic parasitaemia and fever were not seen among children < 2 years of age or adults. The wealth index was not significantly associated with the outcome of fever in either univariate or multivariate analysis, and was not included in the final model.

**Table 1:** Association between parasitaemia and fever

	No parasitaemia	Submicroscopic parasitaemia	Microscopic parasitaemia
Association between parasitaemia and fever^a^			
Age 0.5–< 2			
Risk	37/293 (12.6%)	16/120 (13.3%)	25/71 (35.2%)
RR^b^ (95% CI)	Reference group	1.02 (0.60–1.75)	2.72 (1.71–4.33)
p value	–	0.08	< 0.01
Age 2–< 5			
Risk	88/1158 (7.6%)	59/508 (11.6%)	81/393 (20.6%)
RR^b^ (95% CI)	Reference group	1.42 (1.03–1.98)	2.56 (1.93–3.37)
p value	–	0.03	< 0.01
Age 5–10			
Risk	86/2153 (4.0%)	93/1267 (7.34%)	146/1379 (10.6%)
RR^b^ (95% CI)	Reference group	2.01 (1.49–2.71)	2.93 (2.15–3.99)
p value	–	< 0.01	< 0.01
Age 0.5–10			
Risk	211/3604 (5.6%)	168/1895 (8.9%)	252/1843 (13.7%)
RR^b^ (95% CI)	Reference group	1.67 (1.37–2.03)	2.64 (2.16–3.22)
p value	–	< 0.01	< 0.01
Age ≥ 18			
Risk	50/948 (5.2%)	48/679 (7.1%)	8/106 (7.6%)
RR^b^ (95% CI)	Reference group	1.31 (0.23–0.85)	1.47 (0.70–3.06)
p value	–	0.23	0.31

	No parasitaemia	Submicroscopic parasitaemia
Association between parasitaemia and fever, excluding those with recent malaria^c^		
Age 0.5–< 2		
Risk	37/284 (13.0%)	14/81 (17.3%)
RR^b^ (95% CI)	Reference group	1.32 (0.76–2.31)
p value	–	0.33
Age 2–< 5		
Risk	84/1100 (7.6%)	47/378 (12.4%)
RR^b^ (95% CI)	Reference group	1.52 (1.07–2.17)
p value	–	0.02
Age 5–10		
Risk	79/2034 (3.9%)	89/1161 (7.7%)
RR^b^ (95% CI)	Reference group	1.97 (1.48–2.63)
p value	–	< 0.01
Age 0.5–10		
Risk	200/3418 (5.9%)	150/1620 (9.3%)
RR^b^ (95% CI)	Reference group	1.64 (1.34–2.01)
p value	–	< 0.01
Age ≥ 18		
Risk	48/930 (5.2%)	47/672 (7.0%)
RR^b^ (95% CI)	Reference group	1.31 (0.85–2.05)
p value	–	0.23

^a^Reported fever in prior 24 h or documented temperature of ≥ 38.0 °C at routine visit

^b^Adjusted for repeated measures in the same study participant

^c^Excludes participants who were diagnosed with malaria in the past 14 days or developed malaria in the next 7 days

As malarial fevers are typically intermittent, the usual definition for malaria diagnosis includes either history of fever or documented fever. However, documented fevers were considered as an alternative, more specific outcome (see Additional file 1). There were smaller numbers for this outcome, which did not allow for age stratification, but the findings were similar to those when all fevers were considered. There was again a significant association between microscopic parasitaemia and documented fever in children, compared to no parasitaemia, with a > fivefold increase in risk. Among children, having submicroscopic parasitaemia compared to no parasitaemia was associated with an increased risk of documented fever (aRR [CI], 2.08 [1.18–3.66]), and this association was maintained when those with recent malaria were excluded from the analysis (aRR [CI], 1.87 [1.03–3.37]). Neither microscopic nor submicroscopic parasitaemia was associated with increased risk of documented fever in adults.

### Association of parasitaemia and non-febrile clinical illness

In children of all ages, microscopic parasitaemia was associated with an increased risk of non-febrile clinical illness, compared to those without parasitaemia (Table 2). However, submicroscopic parasitaemia, compared to no parasitaemia was associated with non-febrile clinical illness only in children aged 5–10 years (aRR [CI] 1.44 [1.17–1.78]). The association in this age group was maintained when excluding those with recent malaria. Wealth index was not significantly associated with the outcome of non-febrile clinical illness in either univariate or multivariate analysis, and was not included in the final model. For adults, neither microscopic nor submicroscopic parasitaemia was associated with increased risk of non-febrile clinical illness.

**Table 2:** Association between parasitaemia and non-febrile clinical illness

	No parasitaemia	Submicroscopic parasitaemia	Microscopic parasitaemia
Association between parasitaemia and non-febrile clinical illness^a^			
Age 0.5–< 2			
Risk	74/293 (25.3%)	27/120 (22.5%)	31/71 (43.7%)
RR^b^ (95% CI)	Reference group	0.90 (0.60–1.36)	1.73 (1.28–2.35)
p value	–	0.60	< 0.01
Age 2–< 5			
Risk	181/1158 (15.6%)	96/508 (18.9%)	87/393 (22.1%)
RR^b^ (95% CI)	Reference group	1.16 (0.92–1.46)	1.39 (1.09–1.77)
p value	–	0.22	0.01
Age 5–10			
Risk	185/2153 (8.6%)	152/1267 (12.0%)	191/1379 (13.9%)
RR^b^ (95% CI)	Reference group	1.44 (1.17–1.78)	1.71 (1.37–2.13)
p value	–	< 0.01	< 0.01
Age 0.5–10			
Risk	440/3604 (12.2%)	275/1895 (14.5%)	309/1843 (16.8%)
RR^b^ (95% CI)	Reference group	1.26 (1.09–1.47)	1.52 (1.30–1.78)
p value	–	< 0.01	< 0.01
Age ≥ 18			
Risk	119/948 (12.6%)	94/679 (13.8%)	12/106 (11.3%)
RR^b^ (95% CI)	Reference group	1.10 (0.82–1.48)	0.96 (0.52–1.76)
p value	–	0.52	0.89

	No parasitaemia	Submicroscopic parasitaemia
Association between parasitaemia and non-febrile clinical illness, excluding those with recent malaria^c^		
Age 0.5–< 2		
Risk	72/284 (25.4%)	22/81 (27.2%)
RR^b^ (95% CI)	Reference group	1.08 (0.70–1.66)
p value	–	0.74
Age 2–< 5		
Risk	172/1100 (15.6%)	72/378 (19.1%)
RR^b^ (95% CI)	Reference group	1.18 (0.90–1.53)
p value	–	0.23
Age 5–10		
Risk	175/2034 (8.6%)	144/1161 (12.4%)
RR^b^ (95% CI)	Reference group	1.45 (1.16–1.80)
p value	–	< 0.01
Age 0.5–10		
Risk	419/3418 (12.3%)	238/1620 (14.7%)
RR^b^ (95% CI)	Reference group	1.25 (1.07–1.46)
p value	–	< 0.01
Age ≥ 18		
Risk	117/930 (12.6%)	93/672 (13.8%)
RR^c^ (95% CI)	Reference group	1.10 (0.82–1.48)
p value	–	0.53

^a^Composite variable based on self-report of abdominal pain, anorexia, vomiting, diarrhea, cough, headache, joint pain, muscle ache, seizure, or jaundice at routine visit

^b^Adjusted for repeated measures in the same study participant

^c^Excludes participants who were diagnosed with malaria in the past 14 days or developed malaria in the next 7 days

## Discussion

It is increasingly appreciated that submicroscopic malaria parasitaemia is common in both high- and low-transmission settings, but the clinical implications of low-level parasitaemia remain unclear [6, 15–17]. This study assessed relevant data from repeated surveys in a cohort of Ugandan children and adults, followed over 5 years, to measure associations between parasitaemia and clinical outcomes. The associations between microscopic parasitaemia and clinical outcomes in children in this study were expected; malaria is, by definition, microscopic parasitaemia plus clinical symptoms. More notably, in children submicroscopic parasitaemia was associated with an increased risk of both fever and non-febrile illness. These results suggest that submicroscopic infections have clinically relevant consequences for infected children.

Much of the prior work suggesting clinical consequences of submicroscopic parasitaemia has been based on surveys at a single time point, with limited ability to characterize these infections. These studies were limited by inability to distinguish positive molecular results due to low-level active infections, detection of DNA from parasites recently eliminated by treatment, or detection of gametocyte DNA. The study described here benefitted from longitudinal assessment, and therefore the ability to control for recent or subsequent malaria infection.

Data from this study joins a body of work suggesting that even low-density microscopic and submicroscopic infections can be clinically relevant. Clinical consequences of low-density parasitaemia may be due to direct effects of parasitaemia or to immune dysregulation related to infection [18] that increases the risk of non-malarial infections. It is also possible that the observed association does not reflect a causal relationship and that submicroscopic infection is associated with other exposures, though there was no apparent association with wealth. An early study from the Gambia showed association between malaria parasitaemia, including very low-density infections, and non-typhoid Salmonella bacteraemia [19]. A more recent study from Gabon showed that submicroscopic parasitaemia, identified by molecular methods, was present in nearly a quarter of all febrile, blood smear negative children surveyed, although the study design did not allow for comparison of parasite prevalence in non-febrile children [20].

In this study’s cohort, age was a powerful modifying factor for the association between parasitaemia and clinical symptoms. Young children (< 2 years old) had an expected association between microscopically detected parasitaemia and both fever and non-febrile illness. However, in contrast to older children, in these young children submicroscopic infection was not associated with either febrile or non-febrile illness. Low numbers of children in this age group may have prevented the identification of an association, but it is possible that the persistence of fetal haemoglobin, or other age-related differences in the immune system of infants protected against clinical effects of low-density infections [21, 22]. In older children, significant associations were seen between submicroscopic parasitaemia and fever in children aged 2–< 5 and 5–10 years, and between submicroscopic parasitaemia and non-febrile illness in children aged 5–10 years. These results are consistent with recent findings from Rwanda, where children aged 6–10 with submicroscopic infection had more frequent fever, tiredness, weakness, poor appetite, and vomiting than among their uninfected peers [23]. It is worth noting that submicroscopic infection is very common in school-aged children in many malaria-endemic areas, [24, 25], so even modest associations with clinical consequences may lead to important morbidity in this population. In adults, neither microscopic nor submicroscopic parasitaemia was associated with an increased risk of fever or non-febrile clinical illness, presumably due to effective but non-sterilizing immunity.

The concept that submicroscopic infection has clinical consequences strengthens arguments for malaria control strategies designed to eliminate all malaria parasitaemia. These strategies may include mass drug administration, which has been used in Africa intermittently for decades [26, 27], or mass and focal screen and treatment strategies, which are being studied in Africa and South East Asia [28–30]. Availability of high-sensitivity diagnostics including high-sensitivity RDTs could improve the efficacy of these efforts, and awareness that individual children may see a health benefit from treating their low-density infections may enhance acceptance of such strategies by clinicians and local communities. Relevant to targeting of these infections, treatment of chronic asymptomatic infection in a setting with seasonal transmission did not appear to increase risk of clinical malaria during subsequent transmission seasons [31].

## Conclusions

This study demonstrates a compelling association between submicroscopic malaria infection and clinical illness in children in a high-burden country. These findings highlight the importance of defining the submicroscopic malaria parasite reservoir, provide evidence for individual benefit from treatment of low-density malaria parasitaemia, and support aggressive interventions geared to eliminate submicroscopic malaria infections.

## Additional file

**Additional file 1.** Association between parasitaemia and documented fever.

## References

[1] WHO. World malaria report 2015. Geneva: World Health Organization; 2015. http://apps.who.int/iris/bitstream/10665/200018/1/9789241565158_eng.pdf.

[2] Imwong M, Nguyen TN, Tripura R, Peto TJ, Lee SJ, Lwin KM (2015). The epidemiology of subclinical malaria infections in South-East Asia: findings from cross-sectional surveys in Thailand–Myanmar border areas, Cambodia, and Vietnam. Malar J.

[3] Imwong M, Stepniewska K, Tripura R, Peto TJ, Lwin KM, Vihokhern B (2016). Numerical distributions of parasite densities during asymptomatic malaria. J Infect Dis.

[4] Rek J, Katrak S, Obasi H, Nayebare P, Katureebe A, Kakande E (2016). Characterizing microscopic and submicroscopic malaria parasitaemia at three sites with varied transmission intensity in Uganda. Malar J.

[5] Gaye A, Bousema T, Libasse G, Ndiath MO, Konaté L, Jawara M (2015). Infectiousness of the human population to *Anopheles arabiensis* by direct skin feeding in an area hypoendemic for malaria in Senegal. Am J Trop Med Hyg.

[6] Ouédraogo AL, Gonçalves BP, Gnémé A, Wenger EA, Guelbeogo MW, Ouédraogo A (2015). Dynamics of the human infectious reservoir for malaria determined by mosquito feeding assays and ultrasensitive malaria diagnosis in Burkina Faso. J Infect Dis.

[7] Chen I, Clarke SE, Gosling R, Hamainza B, Killeen G, Magill A (2016). “Asymptomatic” malaria: a chronic and debilitating infection that should be treated. PLoS Med.

[8] de Mast Q, Brouwers J, Syafruddin D, Bousema T, Baidjoe AY, de Groot PG (2015). Is asymptomatic malaria really asymptomatic? Hematological, vascular and inflammatory effects of asymptomatic malaria parasitemia. J Infect.

[9] Okello PE, Van Bortel W, Byaruhanga AM, Correwyn A, Roelants P, Talisuna A (2006). Variation in malaria transmission intensity in seven sites throughout Uganda. Am J Trop Med Hyg.

[10] Kamya MR, Arinaitwe E, Wanzira H, Katureebe A, Barusya C, Kigozi SP (2015). Malaria transmission, infection, and disease at three sites with varied transmission intensity in Uganda: implications for malaria control. Am J Trop Med Hyg.

[11] Katureebe A, Zinszer K, Arinaitwe E, Rek J, Kakande E, Charland K (2016). Measures of malaria burden after long-lasting insecticidal net distribution and indoor residual spraying at three sites in Uganda: a prospective observational study. PLoS Med.

[12] Plowe CV, Djimde A, Bouare M, Doumbo O, Wellems TE (1995). Pyrimethamine and proguanil resistance-conferring mutations in *Plasmodium falciparum* dihydrofolate reductase: polymerase chain reaction methods for surveillance in Africa. Am J Trop Med Hyg.

[13] Polley SD, Mori Y, Watson J, Perkins MD, González IJ, Notomi T (2010). Mitochondrial DNA targets increase sensitivity of malaria detection using loop-mediated isothermal amplification. J Clin Microbiol.

[14] Tusting LS, Rek JC, Arinaitwe E, Staedke SG, Kamya MR, Bottomley C (2016). Measuring socioeconomic inequalities in relation to malaria risk: a comparison of metrics in rural Uganda. Am J Trop Med Hyg.

[15] Bonnet S, Gouagna LC, Paul RE, Safeukui I, Meunier JY, Boudin C (2003). Estimation of malaria transmission from humans to mosquitoes in two neighbouring villages in south Cameroon: evaluation and comparison of several indices. Trans R Soc Trop Med Hyg.

[16] Bousema T, Dinglasan RR, Morlais I, Gouagna LC, van Warmerdam T, Awono-Ambene PH (2012). Mosquito feeding assays to determine the infectiousness of naturally infected *Plasmodium falciparum* gametocyte carriers. PLoS ONE.

[17] Ouédraogo AL, Bousema T, Schneider P, de Vlas SJ, Ilboudo-Sanogo E, Cuzin-Ouattara N (2009). Substantial contribution of submicroscopical *Plasmodium falciparum* gametocyte carriage to the infectious reservoir in an area of seasonal transmission. PLoS ONE.

[18] Langhorne J, Ndungu FM, Sponaas A-M, Marsh K (2008). Immunity to malaria: more questions than answers. Nat Immunol.

[19] Mabey DC, Brown A, Greenwood BM (1987). *Plasmodium falciparum* malaria and Salmonella infections in Gambian children. J Infect Dis.

[20] Mawili-Mboumba DP, Ndong RN, Rosa NB, Largo JLL, Lembet-Mikolo A, Nzamba P (2017). Submicroscopic falciparum malaria in febrile individuals in urban and rural areas of Gabon. Am J Trop Med Hyg.

[21] Dechavanne C, Cottrell G, Garcia A, Migot-Nabias F (2015). Placental malaria: decreased transfer of maternal antibodies directed to *Plasmodium falciparum* and impact on the incidence of febrile infections in infants. PLoS ONE.

[22] Dobbs KR, Dent AE (2016). Plasmodium malaria and antimalarial antibodies in the first year of life. Parasitology.

[23] Sifft KC, Geus D, Mukampunga C, Mugisha JC, Habarugira F, Fraundorfer K (2016). Asymptomatic only at first sight: malaria infection among schoolchildren in highland Rwanda. Malar J.

[24] Sumari D, Mwingira F, Selemani M, Mugasa J, Mugittu K, Gwakisa P (2017). Malaria prevalence in asymptomatic and symptomatic children in Kiwangwa, Bagamoyo district, Tanzania. Malar J.

[25] Walldorf JA, Cohee LM, Coalson JE, Bauleni A, Nkanaunena K, Kapito-Tembo A (2015). School-age children are a reservoir of malaria infection in Malawi. PLoS ONE.

[26] Newby G, Hwang J, Koita K, Chen I, Greenwood B, von Seidlein L (2015). Review of mass drug administration for malaria and its operational challenges. Am J Trop Med Hyg.

[27] Poirot E, Skarbinski J, Sinclair D, Kachur SP, Slutsker L, Hwang J (2013). Mass drug administration for malaria. Cochrane Database Syst Rev.

[28] Hoyer S, Nguon S, Kim S, Habib N, Khim N, Sum S (2012). Focused Screening and Treatment (FSAT): a PCR-based strategy to detect malaria parasite carriers and contain drug resistant *P. falciparum*, Pailin, Cambodia. PLoS ONE.

[29] Larsen DA, Bennett A, Silumbe K, Hamainza B, Yukich JO, Keating J (2015). Population-wide malaria testing and treatment with rapid diagnostic tests and artemether–lumefantrine in southern Zambia: a community randomized step-wedge control trial design. Am J Trop Med Hyg.

[30] Samuels AM, Awino N, Odongo W, Abong’o B, Gimnig J, Otieno K (2017). Community-based intermittent mass testing and treatment for malaria in an area of high transmission intensity, western Kenya: study design and methodology for a cluster randomized controlled trial. Malar J.

[31] Portugal S, Tran TM, Ongoiba A, Bathily A, Li S, Doumbo S (2017). Treatment of chronic asymptomatic *Plasmodium falciparum* infection does not increase the risk of clinical malaria upon reinfection. Clin Infect Dis.

