# Supplementary material for: Clinical consequences of submicroscopic malaria parasitaemia in Uganda
Source: Malar J. 2018 Feb 5;17:67. doi: 10.1186/s12936-018-2221-9 (PMC5800031; doi:10.1186/s12936-018-2221-9)
Supplement: Supplementary file 1 — Additional file 1. Association between parasitaemia and documented fever. [file 12936_2018_2221_MOESM1_ESM.docx]

**Additional File 1. Association between parasitaemia and documented fever**

**Association between parasitaemia and documented fever^a^**

|  |  | **No parasitaemia** | **Submicroscopic parasitaemia** | **Microscopic parasitaemia** |
| --- | --- | --- | --- | --- |
| **Age 0.5-10** | **Risk** | 26/3,604 (0.7%) | 27/1,895 (1.4%) | 68/1,843 (3.7%) |
|  | **RR^b^ (95% CI)** | Reference group | 2.08 (1.18 – 3.66) | 5.45 (3.29 – 9.02) |
|  | **p-value** | -- | 0.01 | <0.01 |
| **Age > 18** | **Risk** | 1/948 (0.1%) | 2/679 (0.3%) | 1/106 (0.9%) |
|  | **RR^b^ (95% CI)** | Reference group | 2.79 (0.25 – 31.01) | 8.91 (0.55 – 145.3) |
|  | **p-value** | -- | 0.40 | 0.13 |

**Association between parasitaemia documented fever, excluding those with recent malaria^c^**

|  |  | **No parasitaemia** | **Submicroscopic parasitaemia** |
| --- | --- | --- | --- |
| **Age 0.5-10** | **Risk** | 25/3,418 (0.7%) | 22/1,620 (1.4%) |
|  | **RR^b^ (95% CI)** | Reference group | 1.87 (1.03 – 3.37) |
|  | **p-value** | -- | 0.04 |
| **Age > 18** | **Risk** | 1/930 (0.1%) | 2/672 (0.3%) |
|  | **RR^b^ (95% CI)** | Reference group | 2.76 (0.25 – 30.8) |
|  | **p-value** | -- | 0.41 |

^a^ Documented fever of >38.0°C at routine visit

^b^ Adjusted for repeated measures in the same study participant

^c^ Excludes participants who were diagnosed with malaria in the past 14 days or developed malaria in the next 7 days
